# MHC Class II Heterozygosity Associated With Attractiveness of Men and Women

**DOI:** 10.1177/1474704921991994

**Published:** 2021-03-10

**Authors:** Terhi J. Hakkarainen, Indrikis Krams, Vinet Coetzee, Ilona Skrinda, Sanita Kecko, Tatjana Krama, Jorma Ilonen, Markus J. Rantala

**Affiliations:** 1 Department of Biology, Section of Ecology, 8058 University of Turku, Finland; 2 Institute of Ecology and Earth Sciences, University of Tartu, Estonia; 3 Institute of Life Sciences and Technologies, 87146 Daugavpils University, Latvia; 4 Department of Zoology and Animal Ecology, Faculty of Biology, University of Latvia, Rīga, Latvia; 5 Department of Genetics, 56410University of Pretoria, Hatfield, South Africa; 6 Daugavpils Regional Hospital, Daugavpils, Latvia; 7 Immunogenetics Laboratory, Institute of Biomedicine, University of Turku, Finland; 8 Clinical Microbiology, Turku University Hospital, Finland

**Keywords:** major histocompatibility complex, human leucocyte antigen, heterozygosity, attractiveness, adiposity, human mate choice

## Abstract

The genes of the Major Histocompatibility Complex (MHC), which plays a fundamental role in the immune system, are some of the most diverse genes in vertebrates and have been connected to mate choice in several species, including humans. While studies suggest a positive relationship between MHC diversity and male facial attractiveness, the connection of MHC diversity to other visual traits and female attractiveness is still unclear. The purpose of this study was to investigate further whether MHC heterozygosity, indicating genetic quality, is associated with visual traits affecting mate preferences in humans. In total 74 Latvian men and 49 women were genotyped for several MHC loci and rated for facial and, in men, also body attractiveness. The results indicate a preference for MHC heterozygous female and male faces. However, the initially positive relationship between MHC heterozygosity and facial attractiveness becomes non-significant in females, when controlling for multiple testing, and in males, when age and fat content is taken into account, referring to the importance of adiposity in immune function and thus also attractiveness. Thus overall the effect of MHC heterozygosity on attractiveness seems weak. When considering separate loci, we show that the main gene related to facial attractiveness is the MHC class II DQB1; a gene important also in viral infections and autoimmune diseases. Indeed, in our study, heterozygous individuals are rated significantly more attractive than their homozygous counterparts, only in relation to gene DQB1. This study is the first to indicate a link between DQB1 and attractiveness in humans.

## Introduction

By choosing mates with traits that indicate “good genes”, individuals might gain several benefits. Such preferences are thought to be adaptive as the “good genes” from the chosen mates pass on to the offspring raising their attractiveness (Fisher, 1915) or viability (Hamilton & Zuk, 1982). The traits selected include visual, vocal and olfactory signals and the cues reflecting indirect benefits for the offspring and also direct benefits in the form of parental care and resources (Kirkpatrick & Ryan, 1991). One of the genetic regions connected to mate choice in humans, as well as many other vertebrates, is the major histocompatibility complex (MHC, termed human leucocyte antigen, HLA, in humans), which protein products play a fundamental role in vertebrate immune processes (Havlicek & Roberts, 2009; Klein & Sato, 2000b; Penn & Potts, 1999).

The MHC genes involved in the immune response are divided into two classes: MHC class I genes (e.g. HLA-A, B and C) coding for the α-polypeptide chain of the class I molecule and MHC class II genes (e.g. HLA-DQB1, DQA1 and DRB1) coding for the α- and β-polypeptide chains of the class II molecules (Klein & Sato, 2000b). Genes belonging to MHC class I are expressed in almost all somatic cells; MHC class II genes, on the other hand, are in normal conditions expressed only in a subgroup of immune cells including B-cells, activated T-cells, macrophages, dendritic cells and thymic epithelial cells called as a group also antigen presenting cells (Howard, 1987). Both MHC class I and MHC class II molecules function as initiators of the adaptive immune response by presentation of short antigen derived peptides to T-cells, which develop into cytotoxic T-cells or helper T-cells (Klein & Sato, 2000b). When activated by MHC I–peptide complex, cytotoxic T-cells are capable of killing antigen presenting cell and thus limit the spread of intracellular pathogens like viral infections. Helper T-cells, on the other hand, become activated mainly by peptides derived from molecules phagocytosed by the antigen presenting cell and presented by MHC II molecule, and fight the infections by further activating macrophages, B cells and cytotoxic T-cells (Klein & Sato, 2000b).

Most of the genes in the MHC region express extremely high intrapopulation polymorphism, which has been explained by different kinds of balancing selection, including pathogen driven selection, heterozygote advantage and sexual selection (Apanius et al., 1997; Brown & Eklund, 1994; Havlicek & Roberts, 2009). In pathogen driven frequency dependent selection models, MHC polymorphism has been thought to be a consequence of an evolutionary arms race between the pathogens and vertebrates (Havlicek & Roberts, 2009), although also the spatial and temporal variation of pathogens, would lead to overall higher fitness of heterozygous compared homozygous individuals, and thus would maintain the intrapopulation polymorphism (Hedrick, 2002). The heterozygote advantage, which we focus on, can be explained through the function of MHC genes: because of codominant expression of the MHC genes, a larger number of peptides stimulating immune response can be presented to T cells in MHC heterozygous individuals, compared to MHC homozygous individuals (Havlicek & Roberts, 2009). The advantageous heterozygosity in the MHC area can be maintained, in addition to natural selection, by sexual selection. Mate preferences favoring heterozygotes and rare alleles or genetically dissimilar individuals, would lead to increased offspring heterozygosity (Havlicek & Roberts, 2009; Mitton et al., 1993), and thus preferences based on partner’s MHC diversity deserve to be more thoroughly explored (Radwan et al., 2020). Although the favoring of genetically dissimilar individuals might be a part of general inbreeding avoidance to prevent the effect of recessive deleterious alleles (Apanius et al., 1997; Penn & Potts, 1999), it has been shown that selection for MHC diversity can be independent of overall genomic diversity (Lie et al., 2008). By favoring MHC heterozygous individuals instead of MHC dissimilar individuals, one would avoid extreme outbreeding and might achieve optimal rather than maximal heterozygosity in the offspring (Bateson, 1980; Nowak et al., 1992). Indeed, avoiding extreme outbreeding might cause the higher fitness found in some distantly related human couples (Helgason et al., 2008), while at least in sticklebacks, optimal MHC heterozygosity compared to maximal MHC heterozygosity is connected to lower parasitic load, and thus better survival (Milinski, 2003). This reproductive pattern supports the “good genes as heterozygosity” -hypothesis presented by Brown (Brown, 1998), which suggest that mate choice, in relation to MHC, should operate in a way that provides the offspring the best possible immune defense, and thus improves their fitness. Optimizing the MHC heterozygosity of the offspring would be profitable, while heterozygosity seems to indicate better immune response, compared to MHC homozygosity, by for example enhancing resistance to diseases such as Hepatitis B (Thursz et al., 1997) and delaying the onset to AIDS in HIV-1-infections (Carrington et al., 1999). In addition, favoring of MHC heterozygous mates might also mean direct benefits, while the MHC heterozygous mates selected could have a reduced risk of transmitting diseases provided by more effective immunity (Lie et al., 2008). Although, when very attractive, mates might have had more sexual encounters and thus might carry more sexually transmitted diseases (Fethers et al., 2008; Zaromatidis et al., 2004).

Reflecting both indirect and direct benefits of favoring MHC heterozygous mates, MHC heterozygosity has indeed been connected to higher reproductive success and better advertisement of sexually selected male traits, compared to MHC homozygous individuals, in several mammal as well as bird species (Ditchkoff et al., 2001; Sauermann et al., 2001; Seddon et al., 2004). In primates, selection for MHC diverse males occurs in several species, including rhesus macaque (*Macaca mulatta*), grey mouse lemur (*Microcebus murinus*), fat-tailed dwarf lemur (*Cheirogaleus medius*), mandrill (*Mandrillus sphinx*) (Setchell & Huchard, 2010) and humans (Havlicek & Roberts, 2009; Winternitz & Abbate, 2015). In humans, studies have mostly concentrated on MHC-related mate preferences in olfactory cues and mate choice in actual couples. The results from MHC-related mate choice studies made on established couples, such as married couples, are very mixed: some show a bias toward MHC similarity (Rosenberg et al., 1983), some dissimilarity (Garver-Apgar et al., 2006; Ober et al., 1997) and some to random distribution (Ihara et al., 2000; Jin et al., 1995; Nordlander et al., 1983). Odor-based MHC studies, on the other hand, have mostly revealed disassortative preferences in choosing potential partners (Thornhill et al., 2003; Wedekind & Furi, 1997; Wedekind et al., 1995). Although facial preferences arise early in development and across cultures, only recently has facial attractiveness been connected to the MHC region (Havlicek & Roberts, 2009; Rhodes, 2006). A few facial attractiveness studies made, mostly indicate a preference for MHC-similar individuals (Havlicek & Roberts, 2009; Roberts, Little, Gosling, Jones, et al., 2005), but also for MHC heterozygous men (Lie et al., 2008; Roberts, Little, Gosling, Perrett, et al., 2005). In an experiment by Roberts et al. (2005) women rated pictures of MHC heterozygous men significantly more attractive than the pictures of MHC homozygous men (Roberts, Little, Gosling, Perrett, et al., 2005). Furthermore, the pictures of skin of heterozygous men were judged healthier than the skin of homozygotes (Roberts, Little, Gosling, Perrett, et al., 2005). This has been thought as an outcome of less pathogen stress on MHC heterozygotes during development, which could contribute to the features affecting attractiveness (Rhodes et al., 2001), such as facial averageness (Lie et al., 2008) and quality of skin (Roberts, Little, Gosling, Perrett, et al., 2005). In females MHC heterozygosity has been shown to be connected to sexual success measured by the number of sexual partners, but not facial attractiveness (Coetzee et al., 2007; Lie et al., 2008, 2010). Finally, it has been shown that preferences related to heterozygosity at the MHC area are independent of the overall preferences for genomic heterozygosity (Carrington et al., 1999; Lie et al., 2008). However, not all studies have confirmed the positive relationship between MHC heterozygosity and facial attractiveness related to mate preferences (Coetzee et al., 2007; Thornhill et al., 2003). In a study of Coetzee et al. (2007) neither HLA heterozygosity nor HLA allele frequency predicted how attractive men rated the female participants. In addition, a study by Thornhill et al. (2003) showed a correlation between MHC heterozygosity and male scent attractiveness, but not between MHC heterozygosity and facial attractiveness in either sex.

The MHC-related preference studies in humans show a great variability in methods and results, but overall indicate a connection between MHC variability and mate preferences. While results from studies made on actual couples seem mixed, and might be more affected by cultural phenomena and expectations than mate preference studies, studies concentrating only on sexually selected traits can reveal preferences important to further improve sexual selection theory in humans. A relationship between MHC genes and odors has been found (Thornhill et al., 2003; Wedekind & Furi, 1997; Wedekind et al., 1995) and explained by MHC linked olfactory receptors (Younger et al., 2001) and the finding that the same peptides that serve as ligands for MHC I molecules, also cause sensory stimuli in the mammalian vomeronasal organ, important for example in mate recognition (Leinders-Zufall et al., 2004). In humans, the odor stimulation of “non-self” or “self” MHC peptides seem to activate areas of frontal cortex and thus the MHC peptides might be detected without the vomeronasal organ, missing from humans, as well (Milinski et al., 2013). Facial attractiveness seems to have a connection to both MHC similarity between women and men (Roberts Little, Gosling, Jones, et al., 2005) and in men to MHC heterozygosity (Lie et al., 2008; Roberts, Little, Gosling, Perrett, et al., 2005; Winternitz & Abbate, 2015). But the mechanism, how MHC genes might be related to visual characters, and if there are differences between the effects of the various genes in the MHC area, is still unclear. Furthermore, no studies have linked MHC heterozygosity to female facial attractiveness or MHC heterozygosity to male body attractiveness (Havlicek & Roberts, 2009; Winternitz & Abbate, 2015), even though, as a visual character, it might also affect mate choice. Certain MHC genes have indeed been shown to affect secondary sexual characters, such as body mass, in other mammals (Ditchkoff et al., 2001).

The purpose of this study was to examine the connection of MHC heterozygosity to visual characters, including facial and body attractiveness, which might affect mate preferences in humans. The level of heterozygosity was determined by genotyping several loci from both MHC class II and MHC class I in a sample of men, and MHC class II loci DQB1 and DQA1 in a sample of women. For males MHC class II genotyping included DQB1, DQA1 and DRB1 loci, which have been studied to define the risk of insulin dependent diabetes mellitus (T1D) (Kiviniemi et al., 2007). The polymorphism of these genes contributes highly to the genetic risk of T1D, but has also been shown to influence mate choice (Kahles et al., 2009). In addition, MHC class I loci HLA-A and HLA-B were genotyped for males, as the molecules encoded by these highly polymorphic genes, found on all nuclear cells, are important in the presentation of microbial antigens to immune cells, eradicating intracellular infections (Klein & Sato, 2000b). For their importance in immunology, these genes have also been studied in multiple mate preference related experiments in males and have shown variable connections to body odor and facial preferences as well as to actual mate choice in marriage (Havlicek & Roberts, 2009).

The main focus of our study was on female mate preferences, because sex differences in parental investment might lead to stronger female than male choice (Trivers, 1972). However, while preferences for MHC diversity, based on studies made so far, have been detectable only in females (Winternitz & Abbate, 2015), we expanded our study to cover also male mate choice in the MHC loci that seemed to be controlling the female mate choice. The focus was on MHC heterozygosity, as it seems to indicate better immune response, compared to MHC homozygosity (Carrington et al., 1999; Thursz et al., 1997) and might thus be associated with physical features selected in mate choice as well (Lie et al., 2008). Based on earlier studies (Carrington et al., 1999; Lie et al., 2008; Thursz et al., 1997), the hypothesis was that MHC heterozygosity would be positively connected to attractiveness.

## Material and Methods

### Collection of Samples

Seventy four Latvian men (mean age = 23.1, SD = 3.9, table S1) and 49 Latvian women (mean age = 20.2, SD = 1.4, table S1) took part in this study. The participants were both staff and students from Daugavpils University and Transportation College of Daugavpils.

Facial photographs and for males also full body photographs were taken from the participants in conditions described in Rantala et al. (2013). Five of the men studied did not give a permission for a body photograph. In addition, each participant’s percentage body fat was measured (Bioelectric impedance analysis, Omron Body Composition Monitor BF-500). The facial and body attractiveness of the males were rated from the male photographs by 94 female students (mean age = 20, SD = 1.89) from the University of Daugavpils from −5 (very unattractive) to +5 (very attractive) and the facial attractiveness of the females was rated by 18 males (mean age = 21.7 years, SD = 1.53) from the University of Daugavpils, in conditions described in Rantala et al. (2013). None of the raters were using hormonal contraception. The faces of full body images were blurred to avoid the use of facial characteristics in body judgments. Inter-rater reliability was high for all ratings (all Cronbach α > 0.93) and thus all the ratings were averaged across raters.

### HLA Genotyping

The blood samples were dried on FTA® Classic Card (Whatman International Ltd., Maidstone, UK) sample collection cards, and genotyping for haplotypes composed of alleles in MHC class II genes HLA-DRB1, -DQA1 and DQB1 was performed as described in detail in Kiviniemi et al. (Kiviniemi et al., 2007). Uncommon haplotypes were further resolved by sequencing for DQB1 by the MegaBace *sequencer* (MB1000), using Nucleo spin Extract II kit (Macherey Nagel) and NucleoSEQ kit (Macherey Nagel) for sample preparation.

The DNA for the HLA class I genotyping was obtained from the blood using a salt extraction protocol similar to that outlined in Aljanabi and Martinez (1997) (Aljanabi & Martinez, 1997). HLA class I genes HLA-A and HLA-B were genotyped with a *LABType*® *SSO* Typing Test using One lambda LABTypes RSSO1A (lot012) and RSSO1B (lot014). The test was also used to genotype 10 unclear individuals for HLA class II gene DR with One lambda LABType RSO2BIT (lot015). For the LABType typing test the concentration of the DNA samples was adjusted with sterile water to 20 ng/µl. The concentration was measured with Qubit® Fluorometer. The results were collected with Luminex 100/200 analyzer and Luminex 100 IS 2.3 Software by using primerspecific templates. The results were analyzed with *HLA Fusion*™ Software Version *2.0*. For every genotyping test we had a subset of positive controls and all unclear samples were genotyped twice. Sufficient results were obtained for all five HLA loci in males, but only two HLA loci (HLA-DQA1 and HLA-DQB1) in females.

### Statistical Analyses

All analyses were performed in SPSS version 26. Overall heterozygosity was calculated in each sex by calculating the proportion of heterozygous HLA loci from all the loci genotyped. Prior to analysis, all variables were examined for accuracy of data entry, missing values, outliers, pairwise linearity and normality of their distributions (Tabachnick & Fidell, 2006). The descriptive statistics of all variables are shown in table S1. All variables were linearly related, except the relationship between percentage fat and body attractiveness in men and the relationship between percentage fat and facial attractiveness in women, in that underweight and overweight individuals were considered less attractive than average weight counterparts (Figure S1, S2). The relationship between percentage fat and facial attractiveness was linear in men. All values were also normally distributed in both sexes (two-tailed critical z score = ±3.29, *p* = 0.001) except overall heterozygosity in the male (skewness z = −6.34, kurtosis z = 5.41) and female (skewness z = −8.28, kurtosis z = 13.84) dataset. Standardized residual plots also indicated that the residuals were not normally distributed. We could not successfully normalize overall heterozygosity in either men or women so Spearman’s correlations and Spearman’s partial correlations with bootstrapping (1,000 iterations) were conducted to test the association between overall heterozygosity and facial attractiveness. Twenty nine of the 74 men studied were partly or totally homozygous for the five HLA loci, while seven of the 42 women were partly or totally homozygous for the two HLA loci. Analysis of Covariance (ANCOVA), with bootsrapping (1,000 iterations), was used to compare the differences between the heterozygous and homozygous groups for the different loci.

## Results

### Overall Heterozygosity and Attractiveness in Males

Overall heterozygosity was significantly and positively correlated with facial attractiveness, indicating that more HLA heterozygous men were considered more attractive (rs = 0.352, N = 74, SE = 0.103, 95% CI = 0.157, 0.545, *p* = 0.002; Table 1). Age and percentage body fat might, however, confound the relationship between HLA heterozygosity and facial attractiveness, since age and body fat were significantly associated with both HLA heterozygosity and facial attractiveness (Table 1): younger and skinnier men were judged more attractive and had higher overall heterozygosity (Table 1). We therefore conducted Spearman’s partial correlations between overall heterozygosity and facial attractiveness, controlling for age and percentage fat, where after the relationship between overall heterozygosity and facial attractiveness was no longer significant (rs = 0.149, df = 70, SE = 0.107, 95% CI = −0.083, 0.353, *p* > 0.1). Heterozygosity was also initially positively correlated with body attractiveness (rs = 0.277, N = 69, SE = 0.111, 95% CI = 0.053, 0.486, *p* = 0.021; Table 1), but not after controlling for age and percentage fat (rs = 0.079, df = 65, SE = 0.099, 95% CI = −0.123, 0.270, *p* > 0.1). Overall, we observed a stronger relationship between overall heterozygosity and facial attractiveness than between overall heterozygosity and body attractiveness in men, although both were non-significant after controlling for age and percentage fat.

**Table 1.** Spearman’s Correlations Between Overall Heterozygosity, Age, Fat Percentage and Facial and Body Attractiveness in Male and Female Participants.

	Overall Heterozygosity	Age	Fat %	Facial Attractiveness	Body Attractiveness
Overall Heterozygosity	—	−0.402***	−0.245*	0.352**	0.277*
Age	−0.009	—	0.342**	−0.511***	−0.477***
Fat %	−0.086	−0.38	—	−0.478***	−0.490***
Facial attractiveness	0.311*	0.006	−0.419***	—	0.554***

*Note:* The results for male participants (N = 74) are indicated above the diagonal and female participants below the diagonal. Both facial and body attractiveness measures were available in men, but only facial attractiveness measurements in women (N = 49). *p ≤ 0.05, **p ≤ 0.01, ***p ≤ 0.001.

### Overall Heterozygosity and Attractiveness in Females

Overall heterozygosity was significantly and positively correlated with facial attractiveness in women, indicating that more HLA heterozygous women were considered more attractive (rs = 0.311, N = 49, SE = 0.107, 95% CI = 0.081, 0.506, *p* = 0.029; Table 1). Percentage fat showed a significant curvilinear association with facial attractiveness (F(2,62) = 15.84, *p* < 0.001, R^2^ = 0.34), in that underweight and overweight women were considered less attractive than average weight women (Figure S2). Age was not significantly correlated with facial attractiveness or overall heterozygosity (Table 1), most likely because the age range in women (18–24) was much smaller than the age range in men (19–31). We therefore conducted Spearman’s partial correlations between overall heterozygosity and facial attractiveness, controlling for percentage fat. The relationship between overall heterozygosity and facial attractiveness was still significant after controlling for percentage fat (rs = 0.291, df = 46, SE = 0.08, 95% CI = 0.138, 0.448, *p* = 0.045), but not after controlling for multiple testing (Bonferroni corrected α = 0.025).

### Heterozygosity at Specific HLA Loci and Attractiveness

#### HLA-DQB1& HLA-DQA1

**Figure 1.**
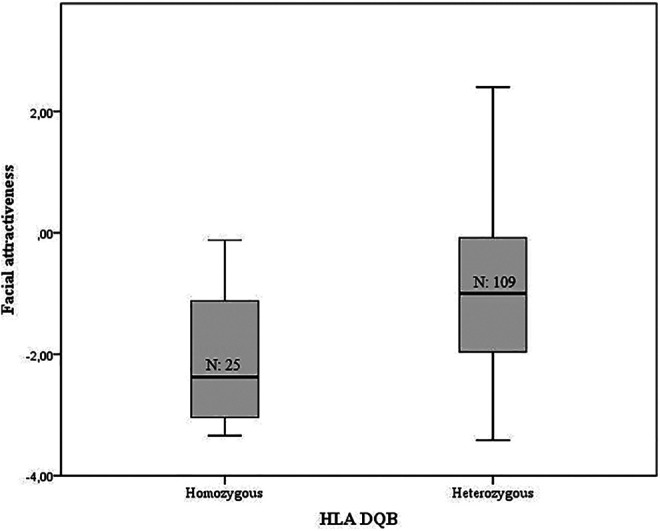
A box plot of facial attractiveness for DQB homozygous and heterozygous individuals when both sexes are combined.

HLA-DQB1 and HLA-DQA1 results were available for both sexes so we conducted an ANCOVA to determine if there is a statistically significant difference in facial attractiveness between heterozygous and homozygous individuals at these loci, controlling for age, sex, and percentage fat. There was a significant effect of HLA-DQB1 on facial attractiveness before (F(1,121) = 10.496, η_p_^2^ = 0.082, p = 0.002, Figure 1) and after controlling for age, sex and percentage fat (F(1,120) = 5.183, η_p_^2^ = 0.045, p = 0.025). There was no significant effect of HLA-DQA1 (F(1,120) = 0.479, η_p_^2^ = 0.004, p > 0.1) or any of their interactions. Heterozygous individuals at the HLA-DQB1 locus were significantly more attractive (M = −0.961, SE = 0.295, 95% CI = −1.439, −0.321) than their homozygous counterparts (M = −1.873, SE = 0.170, 95% CI = −2.195, −1.515) after controlling for age, sex and percentage fat.

We also conducted an ANCOVA to determine if there is a statistically significant difference in body attractiveness between heterozygous and homozygous individuals at these loci, controlling for age and percentage fat (not sex, since body attractiveness ratings were only available for male bodies). Neither HLA-DQB1, nor HLA-DQA1 or their interaction, had a significant effect on body attractiveness before or after controlling for age and percentage fat (p > 0.1).

#### HLA-DRB1, HLA-A and HLA-B

HLA-DRB1, HLA-A and HLA-B results were only available in males so we conducted an ANCOVA to determine if there is a statistically significant difference in facial attractiveness between heterozygous and homozygous individuals at these loci, controlling for age and percentage fat. Neither of these loci, nor any of their interactions, had a significant effect on facial or body attractiveness before or after controlling for age and percentage fat (p > 0.1).

## Discussion

This study provides partial support for the argument that MHC diversity plays a role in male attractiveness (Lie et al., 2008; Roberts, Little, Gosling, Perrett, et al., 2005), by showing positive associations between attractiveness and MHC heterozygosity. In our study of males, we found an initial positive relationship between attractiveness and MHC heterozygosity. However, the positive relationship between MHC diversity and facial, as well as body attractiveness, becomes non-significant when male age and fat percent is considered, implying that age and fat percent have a stronger effect on attractiveness than MHC heterozygosity. Indeed, not all previous studies have evidenced a relationship between MHC heterozygosity and male attractiveness (Coetzee et al., 2007; Thornhill et al., 2003), and thus the positive relationship found between MHC heterozygosity and male attractiveness, might overall be weak and mediated by variables such as adiposity as shown in our data. In previous research, where positive associations between MHC heterozygosity and male facial attractiveness have been found, age and fat percentage have not been controlled for (Lie et al., 2008; Roberts, Little, Gosling, Perrett, et al., 2005) and thus it would be interesting to see if the associations hold after controlling for these parameters.

In our study of females, we found an initial positive relationship between overall MHC heterozygosity and facial attractiveness. However, the relationship becomes non-significant after controlling for multiple testing and thus, overall, the effect of heterozygosity on attractiveness seems weak. A previous study by Lie et al. (2010) have shown a positive relationship between female MHC heterozygosity and sexual success measured by the number of sexual partners, but no relationship between MHC heterozygosity and female facial attractiveness has been found earlier (Coetzee et al., 2007; Lie et al., 2008, 2010). However, earlier studies concentrating on female facial attractiveness and MHC heterozygosity, have been performed in different populations, such as South African Tswana (Coetzee et al., 2007) and Caucasian Australian populations (Lie et al., 2008, 2010), compared to our Latvian study, and thus the results from these studies might not be comparable. It should also be noted that in the previous research, concentrating on female facial attractiveness and MHC heterozygosity, only MHC I loci (Coetzee et al., 2007) or microsatellites in linkage disequilibrium with MHC class I loci A and B or MHC class II locus DR (Lie et al., 2008, 2010) have been studied, while in our study, the females were genotyped for MHC II DQ loci. Thus MHC class II DQ genes are the only MHC genes demonstrated to show a positive relationship between MHC heterozygosity and female facial attractiveness.

In addition to connecting overall MHC heterozygosity weakly to attractiveness, our further analyses showed that heterozygosity in the MHC class II gene DQB1 had the strongest effect on attractiveness in both males and females. From the loci studied, only DQB1 had a significant effect on facial attractiveness, after controlling for age, sex and fat percent, and none of the genes had an effect on male body attractiveness. Indeed, the DQB1 heterozygotes were rated significantly more facially attractive than the DQB1 homozygotes. It should be noted that in previous MHC-related mate choice research, only a few studies (Ihara et al., 2000; Jacob et al., 2002; Ober et al., 1997) have genotyped MHC class II DQ loci, showing allele sharing affecting odor attractiveness (Jacob et al., 2002) and disassortative mating in relation to MHC in couples (Ober et al., 1997), but not an association between MHC heterozygosity and attractiveness. Thus, even though MHC heterozygosity has been connected to male attractiveness earlier (Lie et al., 2008; Roberts, Little, Gosling, Perrett, et al., 2005), this study is the first to study the heterozygosity in MHC class II DQ loci and show a positive relationship between facial attractiveness and heterozygosity in MHC class II DQB1 locus in both sexes. The connection of MHC class II DQB1 locus to facial attractiveness indicates a difference between the effects of the two main MHC class loci on the immune function related to attractiveness. HLA-DQ is a cell surface receptor protein found on antigen presenting cells. It is an αβ heterodimer, where the α and β chains are encoded by two loci, HLA-DQA1 and HLA-DQB1, so it is likely that heterozygosity in the β chain is more closely associated with facial appearance in our population. While foreign antigens derived from pathogens are presented via the DQ protein by phagocytosing cells of immune defense like dendritic cells and macrophages, the helper T-cells are stimulated to proliferate and can signal B-cells to produce antibodies (Klein & Sato, 2000b). But DQ is also involved in presenting common self-antigens and presenting those antigens to the immune system in order to develop tolerance from a very young age. When the tolerance to self proteins is lost, DQ may become involved in autoimmune disease (Klein & Sato, 2000a). MHC class II loci, including DQ, have indeed been connected to the risk of insulin dependent diabetes (Kiviniemi et al., 2007) as well as coeliac disease (Skrabl-Baumgartner et al., 2017) but also protection against hepatitis B virus infection (Thursz et al., 1997). Thus it is possible that heterozygosity in specifically DQB1 locus can affect antibody production and as well be connected to facial attractiveness (Rantala et al., 2013). Furthermore, in male rhesus macaques, DQB1 heterozygosity has been connected to increased reproductive success (Sauermann et al., 2001). In this study MHC class II loci DRB1 and DQA1 did not affect attractiveness nor did heterozygosity at male MHC class I loci, although the differences in the genotyping method between the two MHC classes could have affected the final outcome. While all of the research related to MHC heterozygosity and facial attractiveness, some of which show a positive relationship between MHC heterozygosity and attractiveness in males (Lie et al., 2008; Roberts, Little, Gosling, Perrett, et al., 2005), has concentrated on loci other than DQB1 (Havlicek & Roberts, 2009), it would be interesting to see whether DQB1 shows a connection between MHC heterozygosity and facial attractiveness in other populations as well.

The association between MHC heterozygosity and attractiveness, found in this study, refers to a weak overall association of MHC heterozygosity with visual characters, although one must not rule out the possibility of the mate preferences to be connected to overall genetic diversity instead of MHC diversity. However, some studies have shown that MHC heterozygosity does not correlate with overall genomic heterozygosity and seems to affect mate choice independently (Carrington et al., 1999; Lie et al., 2008). Thus, we can state that this study provides partial support to the “good genes as heterozygosity” -hypothesis suggesting that MHC related mate choice should provide the offspring the best possible immune defense improving their fitness (Brown, 1998). The benefits of optimizing MHC heterozygosity, without extreme outbreeding, can be related to the ability of MHC heterozygotes to present more antigens to T-cells than MHC homozygotes, enhancing the MHC heterozygotes' immune response (Havlicek & Roberts, 2009). While MHC heterozygosity can be inherited, when multiple alleles are considered (Mitton et al., 1993), by selecting attractive mates signaling MHC heterozygosity, one might gain benefits to offspring indirectly via the good genes but also directly via for example high quality resources and reduced risk of transmitted diseases (Fisher, 1915; Kirkpatrick & Ryan, 1991; Lie et al., 2008).

It should also be highlighted that the initially positive relationship between attractiveness and MHC heterozygosity in our male data, is mediated by age and fat percent. While the covariation of age with heterozygosity, is most likely a coincidence arising from the small number of homozygotes, the negative association of fat percent with MHC heterozygosity and attractiveness needs further consideration. Indeed, previous research has shown high body fat proportion to impair attractiveness of both male bodies and faces (Dixson et al., 2007a; Dixson et al., 2007b; Windhager et al., 2011) and adiposity has been shown to play a crucial role in the immune function (Karlsson & Beck, 2010). It has also been shown that antibody production taking place by B-cell derived plasma cells, after interaction between B cells with helper T-cells, can be connected to both adiposity and facial attractiveness (Rantala et al., 2013). Helper T-cells, on the other hand, are activated by the complex formed by the antigen derived peptide presented by MHC class II molecule, either DR, DQ or DP. In general, obesity has been shown to increase the risk for infectious diseases like pneumonia (Baik et al., 2000) and influenza (Louie et al., 2011), through impaired immune function, including a decreased response to antigen stimulation (Karlsson & Beck, 2010). Strongly decreased response to antigen stimulation, would abolish the benefits of heterozygosity and rare alleles in the MHC area. Clearly, more research is needed on the mechanism how fat percent in males is related to MHC gene function and if this mechanism is affected by sex hormones, such as testosterone.

As a conclusion, this study gives partial support to the idea of MHC heterozygosity playing a part in mate choice related to attractiveness. However, in our study, overall MHC heterozygosity is only initially positively related to facial attractiveness in both sexes. In males the relationship between MHC heterozygosity and attractiveness is mostly explained by age and fat percent, which might diminish the benefit of MHC heterozygosity through decreasing immune response (Karlsson & Beck, 2010). In addition, we show that when comparing loci, class II DQB1 has an effect on facial attractiveness possibly linking attractiveness to autoimmune diseases. However, the mechanism, how different MHC genotypes, especially on locus DQB1, might affect visual traits in males and females, and if these patterns are evident across cultures, is still unclear.

## Ethics Statement

The blood sampling was done by professional medical staff in a healthcare center and a written consent was obtained from all the participants. The design of the study was approved by the Research Ethics Committee of Daugavpils University, Latvia. The experiment was conducted according to the principles expressed in the Declaration of Helsinki.

## Supplemental Material

Supplemental Material, sj-pdf-1-evp-10.1177_1474704921991994 - MHC Class II Heterozygosity Associated With Attractiveness of Men and Women Click here for additional data file. Supplemental Material, sj-pdf-1-evp-10.1177_1474704921991994 for MHC Class II Heterozygosity Associated With Attractiveness of Men and Women by Terhi J. Hakkarainen, Indrikis Krams, Vinet Coetzee, Ilona Skrinda, Sanita Kecko, Tatjana Krama, Jorma Ilonen and Markus J. Rantala in Evolutionary Psychology
