# Supplementary material for: MHC Class II Heterozygosity Associated With Attractiveness of Men and Women
Source: Evol Psychol. 2021 Mar 10;19(1):1474704921991994. doi: 10.1177/1474704921991994 (PMC10303478; doi:10.1177/1474704921991994)
Supplement: Supplemental Material, sj-pdf-1-evp-10.1177_1474704921991994 - MHC Class II Heterozygosity Associated With Attractiveness of Men and Women [file sj-pdf-1-evp-10.1177_1474704921991994.pdf]

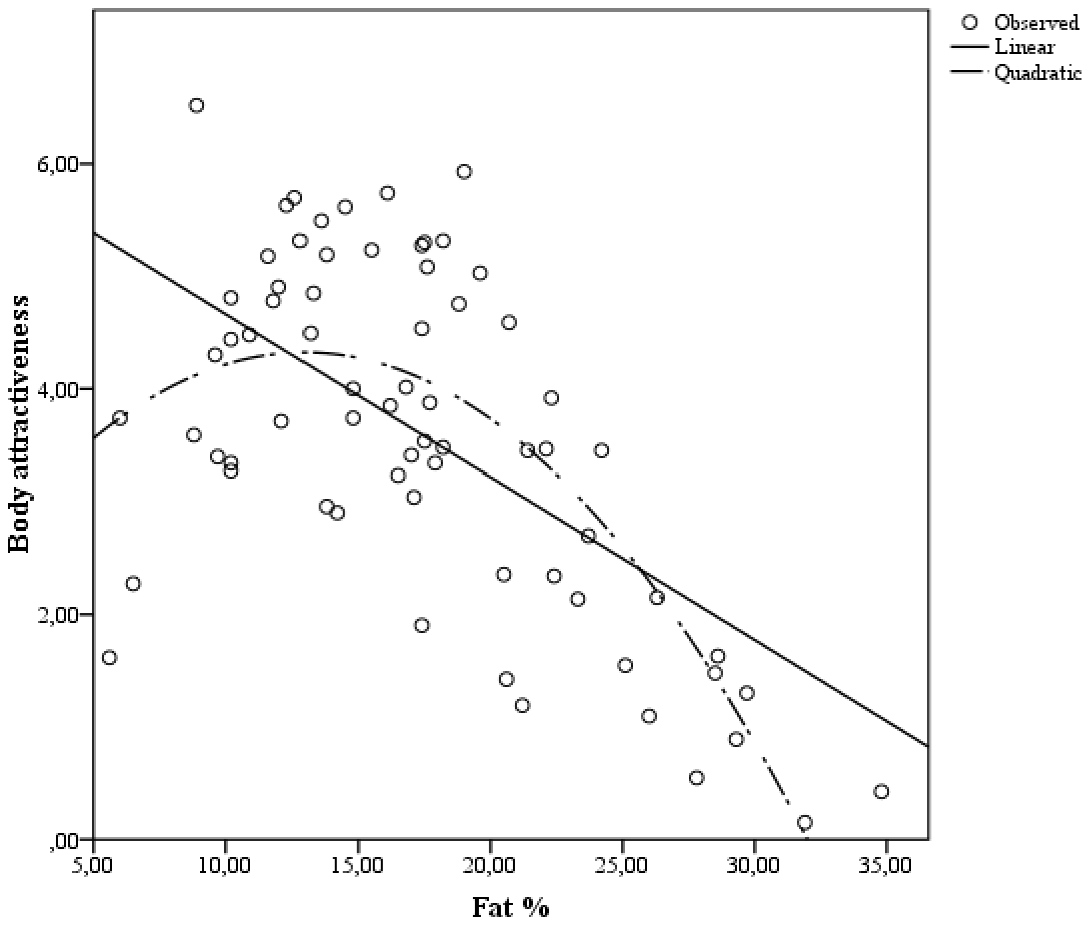

Figure S1. The curvilinear relationship between fat percentage and body attractiveness in men.

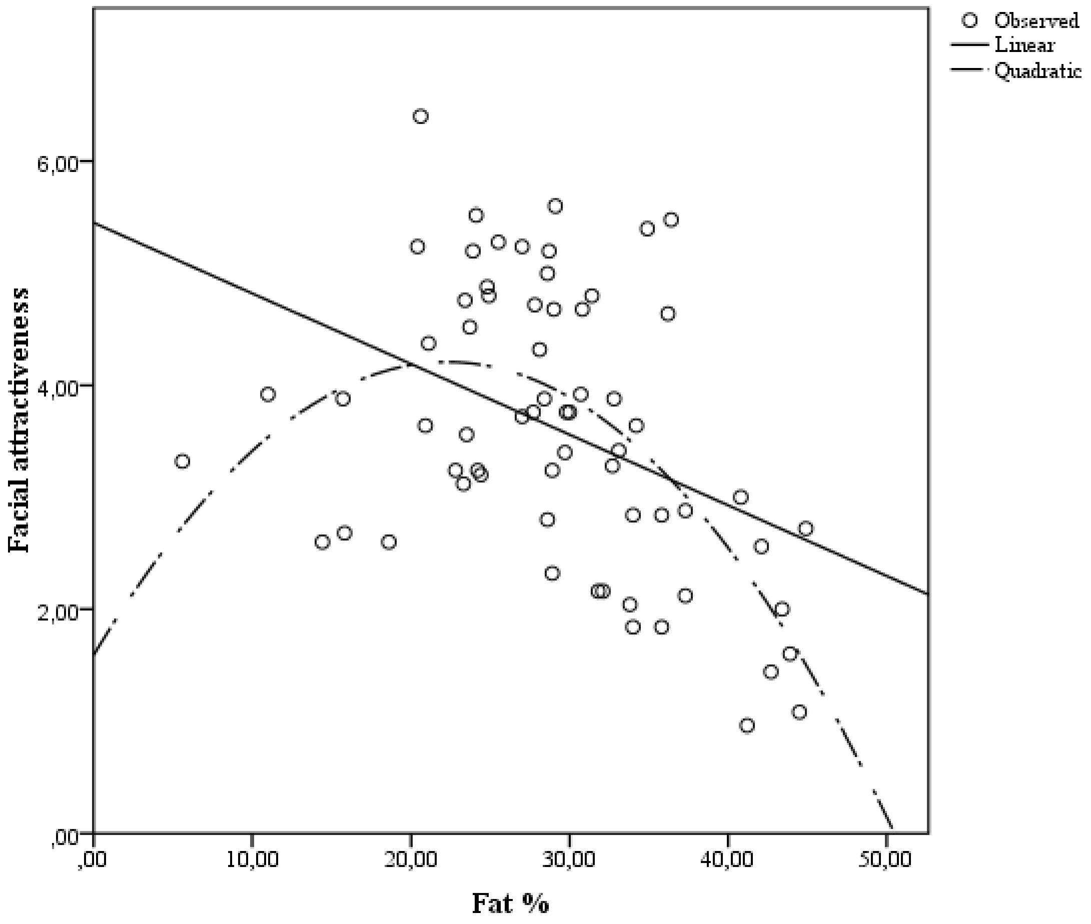

Figure S2. The curvilinear relationship between fat percentage and facial attractiveness in women.

Table S1. Descriptive statistics for the study subjects.

**Variables in males**	Mean (SD)	Min	Max
Heterozygosity	0.85 (0.25)	0.00	1.00
Age	23.12 (3.89)	19.00	31.00
Fat %	17.38 (6.39)	5.60	34.80
Facial attractiveness	-1.78 (1.16)	-3.41	1.78
Body attractiveness	-0.40 (1.55)	-3.85	2.52
**Variables in females**			
Heterozygosity	0.89 (0.29)	0.00	1.00
Age	20.24 (1.38)	18.00	24.00
Fat %	28.67 (7.82)	5.60	44.90
Facial attractiveness	-0.36 (1.18)	-3.04	1.60
